# Supplementary material for: Resolving the full spectrum of human genome variation using Linked-Reads
Source: Genome Res. 2019 Apr;29(4):635–45. doi: 10.1101/gr.234443.118 (PMC6442396; doi:10.1101/gr.234443.118)
Supplement: Supplemental Material [file supp_gr.234443.118_Supplemental_longranger-2.2.zip › longranger-2.2.tar.bz2!/lr2.2-public/lib/stan/cmdstan/stan/doc/api/html/structstan_1_1gm_1_1var__decls__grammar.html]

Stan: stan::gm::var\_decls\_grammar< Iterator > Struct Template Reference


|  |  |
| --- | --- |
|  | Stan  2.5.0  probability, sampling & optimization |


- Main Page
- Related Pages
- Namespaces
- Classes
- Files

- Class List
- Class Index
- Class Hierarchy
- Class Members

All Classes Namespaces Files Functions Variables Typedefs Enumerations Enumerator Friends Macros Pages

- stan
- gm
- var\_decls\_grammar

Public Member Functions |
Public Attributes |
List of all members

stan::gm::var\_decls\_grammar< Iterator > Struct Template Reference

`#include <var_decls_grammar.hpp>`

Inheritance diagram for stan::gm::var\_decls\_grammar< Iterator >:

|  |  |
| --- | --- |
| Public Member Functions | |
|  | var\_decls\_grammar (variable\_map &var\_map, std::stringstream &error\_msgs) |
|  | |

|  |  |
| --- | --- |
| Public Attributes | |
| variable\_map & | var\_map\_ |
|  | |
| std::stringstream & | error\_msgs\_ |
|  | |
| expression\_grammar< Iterator > | expression\_g |
|  | |
| expression07\_grammar< Iterator > | expression07\_g |
|  | |
| boost::spirit::qi::rule  < Iterator,   cholesky\_factor\_var\_decl(var\_origin),   whitespace\_grammar< Iterator > > | cholesky\_factor\_decl\_r |
|  | |
| boost::spirit::qi::rule  < Iterator,   cholesky\_corr\_var\_decl(var\_origin),   whitespace\_grammar< Iterator > > | cholesky\_corr\_decl\_r |
|  | |
| boost::spirit::qi::rule  < Iterator,   corr\_matrix\_var\_decl(var\_origin),   whitespace\_grammar< Iterator > > | corr\_matrix\_decl\_r |
|  | |
| boost::spirit::qi::rule  < Iterator,   cov\_matrix\_var\_decl(var\_origin),   whitespace\_grammar< Iterator > > | cov\_matrix\_decl\_r |
|  | |
| boost::spirit::qi::rule  < Iterator, std::vector  < expression >var\_origin),   whitespace\_grammar< Iterator > > | dims\_r |
|  | |
| boost::spirit::qi::rule  < Iterator, double\_var\_decl(var\_origin),   whitespace\_grammar< Iterator > > | double\_decl\_r |
|  | |
| boost::spirit::qi::rule  < Iterator, std::string(),   whitespace\_grammar< Iterator > > | identifier\_r |
|  | |
| boost::spirit::qi::rule  < Iterator, std::string(),   whitespace\_grammar< Iterator > > | identifier\_name\_r |
|  | |
| boost::spirit::qi::rule  < Iterator, int\_var\_decl(var\_origin),   whitespace\_grammar< Iterator > > | int\_decl\_r |
|  | |
| boost::spirit::qi::rule  < Iterator, matrix\_var\_decl(var\_origin),   whitespace\_grammar< Iterator > > | matrix\_decl\_r |
|  | |
| boost::spirit::qi::rule  < Iterator, std::vector  < expression >var\_origin),   whitespace\_grammar< Iterator > > | opt\_dims\_r |
|  | |
| boost::spirit::qi::rule  < Iterator, ordered\_var\_decl(var\_origin),   whitespace\_grammar< Iterator > > | ordered\_decl\_r |
|  | |
| boost::spirit::qi::rule  < Iterator,   positive\_ordered\_var\_decl(var\_origin),   whitespace\_grammar< Iterator > > | positive\_ordered\_decl\_r |
|  | |
| boost::spirit::qi::rule  < Iterator, range(var\_origin),   whitespace\_grammar< Iterator > > | range\_brackets\_double\_r |
|  | |
| boost::spirit::qi::rule  < Iterator, range(var\_origin),   whitespace\_grammar< Iterator > > | range\_brackets\_int\_r |
|  | |
| boost::spirit::qi::rule  < Iterator,   row\_vector\_var\_decl(var\_origin),   whitespace\_grammar< Iterator > > | row\_vector\_decl\_r |
|  | |
| boost::spirit::qi::rule  < Iterator,   unit\_vector\_var\_decl(var\_origin),   whitespace\_grammar< Iterator > > | unit\_vector\_decl\_r |
|  | |
| boost::spirit::qi::rule  < Iterator, simplex\_var\_decl(var\_origin),   whitespace\_grammar< Iterator > > | simplex\_decl\_r |
|  | |
| boost::spirit::qi::rule  < Iterator, vector\_var\_decl(var\_origin),   whitespace\_grammar< Iterator > > | vector\_decl\_r |
|  | |
| boost::spirit::qi::rule  < Iterator,   boost::spirit::qi::locals  < bool >, var\_decl(bool,   var\_origin),   whitespace\_grammar< Iterator > > | var\_decl\_r |
|  | |
| boost::spirit::qi::rule  < Iterator,   boost::spirit::qi::locals  < bool >, std::vector  < var\_decl >bool, var\_origin),   whitespace\_grammar< Iterator > > | var\_decls\_r |
|  | |

## Detailed Description

### template<typename Iterator> struct stan::gm::var\_decls\_grammar< Iterator >

Definition at line 20 of file var\_decls\_grammar.hpp.

## Constructor & Destructor Documentation

template<typename Iterator >

|  |  |  |  |
| --- | --- | --- | --- |
| var\_decls\_grammar::var\_decls\_grammar | ( | variable\_map & | *var\_map*, |
|  |  | std::stringstream & | *error\_msgs* |
|  | ) |  |  |

Definition at line 653 of file var\_decls\_grammar\_def.hpp.

## Member Data Documentation

template<typename Iterator >

boost::spirit::qi::rule<Iterator, cholesky\_corr\_var\_decl(var\_origin), whitespace\_grammar<Iterator> > stan::gm::var\_decls\_grammar< Iterator >::cholesky\_corr\_decl\_r

Definition at line 48 of file var\_decls\_grammar.hpp.

template<typename Iterator >

boost::spirit::qi::rule<Iterator, cholesky\_factor\_var\_decl(var\_origin), whitespace\_grammar<Iterator> > stan::gm::var\_decls\_grammar< Iterator >::cholesky\_factor\_decl\_r

Definition at line 43 of file var\_decls\_grammar.hpp.

template<typename Iterator >

boost::spirit::qi::rule<Iterator, corr\_matrix\_var\_decl(var\_origin), whitespace\_grammar<Iterator> > stan::gm::var\_decls\_grammar< Iterator >::corr\_matrix\_decl\_r

Definition at line 53 of file var\_decls\_grammar.hpp.

template<typename Iterator >

boost::spirit::qi::rule<Iterator, cov\_matrix\_var\_decl(var\_origin), whitespace\_grammar<Iterator> > stan::gm::var\_decls\_grammar< Iterator >::cov\_matrix\_decl\_r

Definition at line 58 of file var\_decls\_grammar.hpp.

template<typename Iterator >

boost::spirit::qi::rule<Iterator, std::vector<expression>var\_origin), whitespace\_grammar<Iterator> > stan::gm::var\_decls\_grammar< Iterator >::dims\_r

Definition at line 63 of file var\_decls\_grammar.hpp.

template<typename Iterator >

boost::spirit::qi::rule<Iterator, double\_var\_decl(var\_origin), whitespace\_grammar<Iterator> > stan::gm::var\_decls\_grammar< Iterator >::double\_decl\_r

Definition at line 68 of file var\_decls\_grammar.hpp.

template<typename Iterator >

std::stringstream& stan::gm::var\_decls\_grammar< Iterator >::error\_msgs\_

Definition at line 31 of file var\_decls\_grammar.hpp.

template<typename Iterator >

expression07\_grammar<Iterator> stan::gm::var\_decls\_grammar< Iterator >::expression07\_g

Definition at line 36 of file var\_decls\_grammar.hpp.

template<typename Iterator >

expression\_grammar<Iterator> stan::gm::var\_decls\_grammar< Iterator >::expression\_g

Definition at line 34 of file var\_decls\_grammar.hpp.

template<typename Iterator >

boost::spirit::qi::rule<Iterator, std::string(), whitespace\_grammar<Iterator> > stan::gm::var\_decls\_grammar< Iterator >::identifier\_name\_r

Definition at line 78 of file var\_decls\_grammar.hpp.

template<typename Iterator >

boost::spirit::qi::rule<Iterator, std::string(), whitespace\_grammar<Iterator> > stan::gm::var\_decls\_grammar< Iterator >::identifier\_r

Definition at line 73 of file var\_decls\_grammar.hpp.

template<typename Iterator >

boost::spirit::qi::rule<Iterator, int\_var\_decl(var\_origin), whitespace\_grammar<Iterator> > stan::gm::var\_decls\_grammar< Iterator >::int\_decl\_r

Definition at line 83 of file var\_decls\_grammar.hpp.

template<typename Iterator >

boost::spirit::qi::rule<Iterator, matrix\_var\_decl(var\_origin), whitespace\_grammar<Iterator> > stan::gm::var\_decls\_grammar< Iterator >::matrix\_decl\_r

Definition at line 88 of file var\_decls\_grammar.hpp.

template<typename Iterator >

boost::spirit::qi::rule<Iterator, std::vector<expression>var\_origin), whitespace\_grammar<Iterator> > stan::gm::var\_decls\_grammar< Iterator >::opt\_dims\_r

Definition at line 93 of file var\_decls\_grammar.hpp.

template<typename Iterator >

boost::spirit::qi::rule<Iterator, ordered\_var\_decl(var\_origin), whitespace\_grammar<Iterator> > stan::gm::var\_decls\_grammar< Iterator >::ordered\_decl\_r

Definition at line 98 of file var\_decls\_grammar.hpp.

template<typename Iterator >

boost::spirit::qi::rule<Iterator, positive\_ordered\_var\_decl(var\_origin), whitespace\_grammar<Iterator> > stan::gm::var\_decls\_grammar< Iterator >::positive\_ordered\_decl\_r

Definition at line 103 of file var\_decls\_grammar.hpp.

template<typename Iterator >

boost::spirit::qi::rule<Iterator, range(var\_origin), whitespace\_grammar<Iterator> > stan::gm::var\_decls\_grammar< Iterator >::range\_brackets\_double\_r

Definition at line 108 of file var\_decls\_grammar.hpp.

template<typename Iterator >

boost::spirit::qi::rule<Iterator, range(var\_origin), whitespace\_grammar<Iterator> > stan::gm::var\_decls\_grammar< Iterator >::range\_brackets\_int\_r

Definition at line 113 of file var\_decls\_grammar.hpp.

template<typename Iterator >

boost::spirit::qi::rule<Iterator, row\_vector\_var\_decl(var\_origin), whitespace\_grammar<Iterator> > stan::gm::var\_decls\_grammar< Iterator >::row\_vector\_decl\_r

Definition at line 118 of file var\_decls\_grammar.hpp.

template<typename Iterator >

boost::spirit::qi::rule<Iterator, simplex\_var\_decl(var\_origin), whitespace\_grammar<Iterator> > stan::gm::var\_decls\_grammar< Iterator >::simplex\_decl\_r

Definition at line 128 of file var\_decls\_grammar.hpp.

template<typename Iterator >

boost::spirit::qi::rule<Iterator, unit\_vector\_var\_decl(var\_origin), whitespace\_grammar<Iterator> > stan::gm::var\_decls\_grammar< Iterator >::unit\_vector\_decl\_r

Definition at line 123 of file var\_decls\_grammar.hpp.

template<typename Iterator >

boost::spirit::qi::rule<Iterator, boost::spirit::qi::locals<bool>, var\_decl(bool,var\_origin), whitespace\_grammar<Iterator> > stan::gm::var\_decls\_grammar< Iterator >::var\_decl\_r

Definition at line 139 of file var\_decls\_grammar.hpp.

template<typename Iterator >

boost::spirit::qi::rule<Iterator, boost::spirit::qi::locals<bool>, std::vector<var\_decl>bool,var\_origin), whitespace\_grammar<Iterator> > stan::gm::var\_decls\_grammar< Iterator >::var\_decls\_r

Definition at line 145 of file var\_decls\_grammar.hpp.

template<typename Iterator >

variable\_map& stan::gm::var\_decls\_grammar< Iterator >::var\_map\_

Definition at line 30 of file var\_decls\_grammar.hpp.

template<typename Iterator >

boost::spirit::qi::rule<Iterator, vector\_var\_decl(var\_origin), whitespace\_grammar<Iterator> > stan::gm::var\_decls\_grammar< Iterator >::vector\_decl\_r

Definition at line 133 of file var\_decls\_grammar.hpp.

---

The documentation for this struct was generated from the following files:

- src/stan/gm/grammars/var\_decls\_grammar.hpp
- src/stan/gm/grammars/var\_decls\_grammar\_def.hpp


---

[ Stan Home Page ]

*© 2011–2014,
Stan Development Team.*
